# Double- and Multi-Femtosecond Pulses Produced by Birefringent Crystals for the Generation of 2D Laser-Induced Structures on a Stainless Steel Surface

**DOI:** 10.3390/ma12081257

**Published:** 2019-04-17

**Authors:** Fotis Fraggelakis, Giuseppe Giannuzzi, Caterina Gaudiuso, Inka Manek-Hönninger, Girolamo Mincuzzi, Antonio Ancona, Rainer Kling

**Affiliations:** 1 ALPhANOV, Technological Centre for Optics and Lasers, Optic Institute of Aquitaine, rue F. Mitterrand, 33400 Talence, France; girolamo.mincuzzi@alphanov.com (G.M.); rainer.kling@alphanov.com (R.K.); 2 CELIA, University of Bordeaux-CNRS-CEA UMR5107, 33405 Talence, France; inka.manek-honninger@u-bordeaux.fr; 3 Istituto di Fotonica e Nanotecnologie (INF)-CNR U.O.S. Bari, via Amendola 173, I-70126 Bari, Italy; giuseppe.giannuzzi@uniba.it (G.G.); caterina.gaudiuso@uniba.it (C.G.); antonio.ancona@uniba.it (A.A.); 4 Dipartimento Interuniversitario di Fisica, Università degli Studi di Bari, via Amendola 173, I-70126 Bari, Italy

**Keywords:** 2D-LIPSS, metal surface texturing, double pulses, micro/nanostructuring, femtosecond, birefringent crystals

## Abstract

Laser-induced textures have been proven to be excellent solutions for modifying wetting, friction, biocompatibility, and optical properties of solids. The possibility to generate 2D-submicron morphologies by laser processing has been demonstrated recently. Employing double-pulse irradiation, it is possible to control the induced structures and to fabricate novel and more complex 2D-textures. Nevertheless, double-pulse irradiation often implies the use of sophisticated setups for modifying the pulse polarization and temporal profile. Here, we show the generation of homogeneous 2D-LIPSS (laser-induced periodic surface structures) over large areas utilizing a simple array of birefringent crystals. Linearly and circularly polarized pulses were applied, and the optimum process window was defined for both. The results are compared to previous studies, which include a delay line, and the reproducibility between the two techniques is validated. As a result of a systematic study of the process parameters, the obtained morphology was found to depend both on the interplay between fluence and inter-pulse delay, as well as on the number of incident pulses. The obtained structures were characterized via SEM (scanning electron microscopy) and atomic force microscopy. We believe that our results represent a novel approach to surface structuring, primed for introduction in an industrial environment.

## 1. Introduction

Laser surface processing can be employed to generate surface textures of various morphologies, often bio-inspired, characterized by micro-features of different shape, size, dimension, and pattern. Laser-induced textures enable surface functionalities, such as hydrophobicity [1], surface iridescence, coloring [2,3], wear resistance [4], bactericidal activity [5], and selective cell growth [6]. In nature, a systematic link between the surface morphology and functionality is observed [7]. In particular, in the submicron regime, 2D-features found in nature or technically composed exhibit functionalities with great potential for industrial applications, like bactericidal [8] or antireflective [9] properties. Reproducing those morphologies by laser processing can enable such novel surface functionalities on ordinary materials like steel. Nevertheless, the control over the structure morphology is still quite limited in the submicron length scale.

Laser-induced periodic surface structures (LIPSS), the most common laser-induced features in the submicron scale, exhibit usually linear symmetry. Depending on their spatial period, they can be classified as low spatial frequency LIPSS (LSFL) or high spatial frequency LIPSS (HSFL). Typically, the LSFL period is close to the laser wavelength (λ), while the HSFL period is smaller than λ/2 [10]. A significant step forward to control the structure morphologies, in order to fabricate biomimetic textures [11], was achieved with the production of LSFL having 2D symmetry (2D-LIPSS), thanks to the use of different polarization states of the incident radiation [12,13] and to complex irradiation strategies [14]. Double-pulse irradiation (DPI) has shown the formation of a variety of structures in molybdenum [15] and in steel [16,17]. The transformation of 1D-HSFL to 2D-HSFL has been reported applying DPI [18]. The experimental results underline the key role of the inter-pulse delay (Δτ) [19,20] as well as the effect of pulse, wavelength [21], and polarization [22].

In fact, DPI exploits the transient surface state initiated by the first pulse. Depending on the Δτ value, different ultrafast phenomena occur on the surface upon the arrival of the second pulse [23]. For a single femtosecond (fs) laser pulse, the incident light is inhomogeneously absorbed by electrons [24,25], which transfer the energy to the lattice within a few picoseconds via electron–phonon interactions [26]. During this time frame, the material properties, such as electron density, reflectivity, or thermal conductivity, vary [26,27]. The surface temperature increases and after a few tens of picoseconds the expansion of the lattice starts. Depending on the fluence, material melting or ablation will occur within a few tens of picoseconds [28]. Structure formations start in a few hundreds of picoseconds [29]. One of the proposed mechanisms for the generation of LIPSS is the microfluidic movements driven by temperature gradients along the material surface [24]. The occurrence convection flow has been proposed to interpret supra wavelength structures [30], LIPSS [17] and HSFL [31] formation. In this way, DPI processes have proved to enable a tailoring of the surface structure morphologies. However, DPI is often restricted to single spot [19] or single line [15] processing with the use of low repetition lasers and complex setups, preventing its exploitation from large scale applications. Recently, it was shown that the surface morphology can be controlled over large areas by adjusting Δτ, and a variety of structures were produced [17].

In this study, following our previous works on DPI, where we utilized a delay line-based setup [16,17,18], we introduce a robust and easy-to-use setup for double-pulse generation. The setup is combined with a high-repetition-rate laser and a Galvo scanner to texture homogeneously with 2D-LIPSS areas much larger than the spot size. Our setup consists of birefringent crystals of variable length coupled with an industrial high-repetition-rate femtosecond laser to generate double-pulses with different Δτ. Double cross-polarized (XP) and double circularly counter-rotating polarized (CP) pulses were generated in our experiment. A comprehensive study of the process parameters, like fluence (Φ), overlap (pps), and hatch (H), is carried out optimizing the structure homogeneity. The results obtained are compared to our previous study, validating the reproducibility of the process. Moreover, the five used crystals were combined to generate a burst of 32 pulses for surface texturing, which allowed for obtaining novel morphologies. 

## 2. Materials and Methods

**Figure 1:**
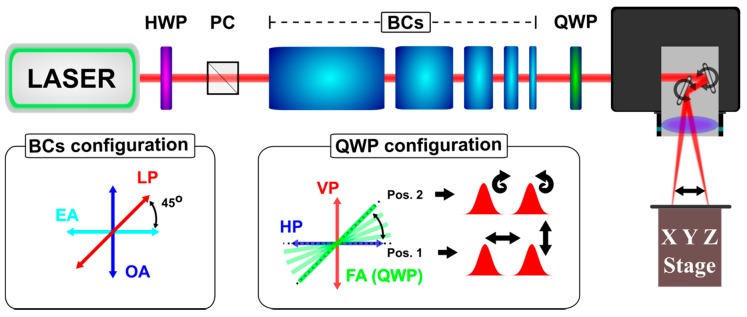
Experimental Setup. **Top**: A set of five removable birefringent crystals (BCs) is used to generate double- or multi-pulses (bursts). The overall BCs configuration shows the used pulse splitting condition—a linearly polarized (LP) laser pulse meets the BCs optical axes (extraordinary axis—EA, ordinary axis—OA) at 45°. A quarter-waveplate (QWP) is utilized to change the double-pulse polarization (vertical polarization—VP, horizontal polarization—HP) from linear to circular, as shown in the QWP configuration frame.

A femtosecond laser, delivering 200-fs pulses at λ = 1030 nm (Pharos from Light Conversion) at a repetition rate of 200 kHz, was utilized for the texturing. Double-pulses with different delays Δτ were generated by splitting the pristine linearly-polarized laser pulse by birefringent crystals (BCs) of different lengths. Within a single crystal, the difference of the refractive index between the extraordinary optical axis (EA) and the ordinary optical axis (OA) and the crystal length determines the Δτ value between the two output pulses. Furthermore, the angle comprised between the polarization vector of the pristine pulse and each optical axis defines the relative intensity of the two pulses. Here this angle was fixed at 45° (Figure 1, BCs configuration), meaning that the intensity and the fluence (the spot size is unchanged) of the two pulses were the same. The scheme of the experimental setup is shown in Figure 1, top. The beam was guided through a sequence of five CaCO_3_ BCs, which can be mounted individually producing double crossed-polarized (XP) pulses. The intensity of the beam was regulated by a rotating half-waveplate (HWP) and a polarizing cube (PC). The used crystals generated inter-pulse delays of Δτ = 1.5 ps, 3 ps, 6 ps, 12 ps, and 24 ps, respectively. The burst containing 32 pulses having Δτ = 1.5 ps was generated by mounting all the crystals simultaneously [32]. By passing through a quarter-wave plate (QWP), in the appropriate angle (Figure 1, QWP configuration), XP or counter-rotating circularly polarized (CP) pulses were obtained [22]. Finally, the beam was delivered on the samples by means of a galvo scanner (intelliSCAN 14 from SCANLAB, Puchheim, Germany) equipped with a 56-mm focal length F-theta lens (Figure 1, top). The spot size (1/e^2^) was 2w_0_ = 24 μm measured by a CMOS camera (FireWire BeamPro Model 2523, Photon Inc., San Jose, CA, USA). During this study, we only processed commercially available, polished, stainless-steel samples (304L) of 0.5-mm thickness, provided by RS Components Ltd., (Corby, UK). Three fluence values were utilized: Φ = Φ_low_ = 0.1 J/cm^2^, Φ = Φ_med_ = 0.2 J/cm^2^, and Φ = Φ_high_ = 0.4 J/cm^2^. The pulse overlapping along a scanning line was determined by the number of pulses per spot delivered (pps) and defined as pps = 2w_0_·f/u, where f is the laser repetition rate and u the scanning speed. The offset between two successive scanning lines was described by the hatch (H). The total pulses per spot on the surface could be estimated by pps_tot_ = (pps·2w_0_)/H and the total dose by d_tot_ = pps_tot_·Φ.

The surface morphology was characterized utilizing a Sigma Field Emission Scanning Electron Microscope (SEM) manufactured by Zeiss (Oberkochen, Germany). The 3D-profiles of the structures were measured via atomic force microscopy (AFM) utilizing a Dimension FastScan manufactured by Bruker (Billerica, MA, USA). Gwyddion software, version 2.46, was used to analyze the SEM images with Fourier transform (FT), and to plot the AFM data and extract the structure dimensions.

## 3. Results and Discussion

### 3.1. The Effect of Delay and Polarization

Within the first few picoseconds after irradiation by the first pulse, the surface undergoes rapid and extreme transformations of its temperature and state [27,33]. Electron relaxation occurring within approximately τ ~ 1 ps [26] and leading to lattice thermalization is the underlining mechanism of the surface transformation [27]. Irradiation with a second pulse, having Δτ in that timescale will be affected by the transient optical properties impacting on the structure formation [17,21,34]. Δτ is considered as the key parameter of the study, since it determines the state of the surface upon which the second pulse will arrive. Apart from Δτ, the pulse fluence [35] and the polarization state [22] are found to have a strong impact on the induced morphology under double-pulse irradiation.

**Figure 2:**
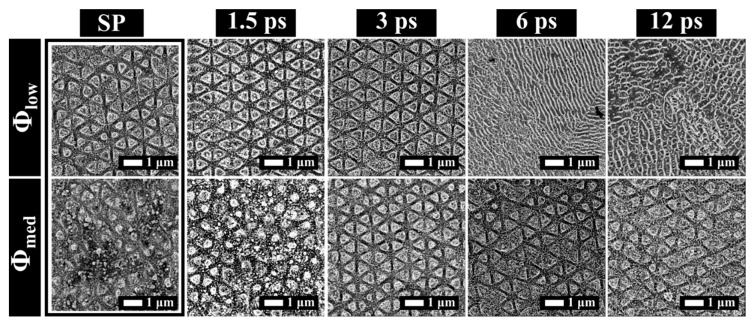
SEM images of stainless steel surface processed with double, counter rotating, and circularly polarized (CP) pulses for different inter-pulse delay values. Two different fluences were utilized Φ_low_ = 0.1 J/cm^2^ (**top**) and Φ_med_ = 0.2 J/cm^2^ (**bottom**). We fixed pps = 20 and H = 2 µm. The results of irradiation with single pulse (SP) trains with circular polarization are included for comparison.

Here we demonstrate the effect of the delay for discrete values of Δτ and for CP and XP pulses. Throughout this part the overlap was fixed to pps = 20, while the offset between scanning lines (H) was fixed to H = 2 µm. In Figure 2, the surface structure morphologies obtained in the case of CP are shown, for Φ_low_ and Φ_med_ versus Δτ. Starting from Φ_low_ (upper row) and single pulse (SP) trains, we observed the formation of triangular structures with a relatively low uniformity. For 1.5 ps ≤ Δτ ≤ 3 ps, the uniformity level increased and periodic structures were observed. The surface morphology for Δτ = 1.5 ps and Δτ = 3 ps consisted of triangular 2D-LIPSS. The process window was in excellent agreement with our previous work utilizing a delay line [17]. A comparison of the optimum process windows between the delay line and BCs setup is shown in Table 1.

By further increasing Δτ (6 ps ≤ Δτ ≤ 12 ps), high spatial frequency LIPSS (HSFL) appeared, showing a period of a few hundreds of nanometers oriented randomly. HSFL formation can be linked to the reduction of the area occupied by low spatial frequency LIPSS (LSFL), in favor of an increase of the HSFL textured area, when 0 ps < Δτ < 5 ps [36]. In detail, as Δτ increased, the irradiated area within which the LSFL threshold was exceeded became smaller for single spot irradiation [36]. When Δτ = 5 ps, the LSFL almost vanished from the surface and HSFL were formed on a titanium surface. Here the transition was observed for 3 ps < Δτ < 6 ps, in agreement with the reported Δτ [36]. This effect is valuable in controlling the hierarchical formation of 2D-LIPSS for scanning over a large area [17]. Lastly, HSFL spatial incoherence can be linked to the lattice orientation in each individual grain [37].

Passing to Φ_med_ for SP and Δτ = 1.5 ps, barely regular 2D structures were formed. The structures obtained a triangular shape, when 3 ps ≤ Δτ ≤ 12 ps. These results not only confirm the important role of the time delay in determining the final structure morphology, but also point out the interplay between the fluence value and Δτ in the 2D-LIPSS formation. In fact, for Φ_low_, triangular structures appeared in correspondence to Δτ values of 1.5 ps ≤ Δτ ≤ 3 ps, which were shorter than for Φ_med_ (3 ps ≤ Δτ ≤ 12 ps). 

**Figure 3:**
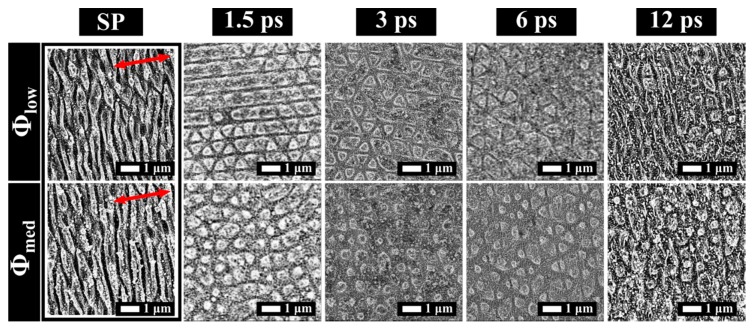
SEM images of stainless steel surface processed with double cross-polarized (XP) pulses for different inter-pulse delay values. Two different fluences were utilized Φ_low_ = 0.1 J/cm^2^ (**top**) and Φ_med_ = 0.2 J/cm^2^ (**bottom**). We fixed pps = 20 and H = 2 µm. The results of irradiation with trains of single pulses (SPs) with linear polarization are included for comparison. The red arrows indicate the polarization direction.

Figure 3 shows the structure morphologies obtained under the same experimental conditions, as in Figure 2 except the pulse polarization state, which are here XP. In all cases, sub-wavelength structures were observed. SP resulted in ripple generation for both Φ_low_ and Φ_med_. In the specific case of Φ_low_, 2D, barely uniform, triangular structures were generated when 1.5 ps ≤ Δτ ≤ 6 ps. Triangular structures disappeared for Δτ = 12 ps. Additionally, for Φ_med_ and 1.5 ps ≤ Δτ ≤ 6 ps 2D, barely uniform structures were generated with a shape evolving from squared (Δτ = 1.5 ps) to quite circular (Δτ = 3 ps) and finally triangular (Δτ = 6 ps). As well as in the case of Φ_low_, also for Φ_med_, triangular structures disappeared for Δτ = 12 ps. By comparing Figure 2 and Figure 3, a remarkable difference between CP and XP pulses was observed, both on the obtained structures and on their uniformity. For example, when Δτ = 1.5 ps and Φ_low_, triangular structures were obtained for CP pulses, whereas for XP pulses the surface was covered by a combination of ripples and triangular structures. When Δτ = 12 ps and Φ_low_, a non-uniform morphology was obtained for double XP pulses and HSFL for CP pulses. The observed differences of the structures obtained with the CP (Figure 2) and the XP (Figure 3) configurations, respectively, can be attributed to the symmetry of the inhomogeneous energy deposition. For linear polarization, surface plasmon polariton (SPP) excitation can strongly impact the interaction for single pulse irradiation [38]. Nevertheless in the case of circular double-pulse irradiation, the elimination of linear symmetry in the induced morphology has been demonstrated experimentally [22] and needs to be further investigated.

**Figure 4:**
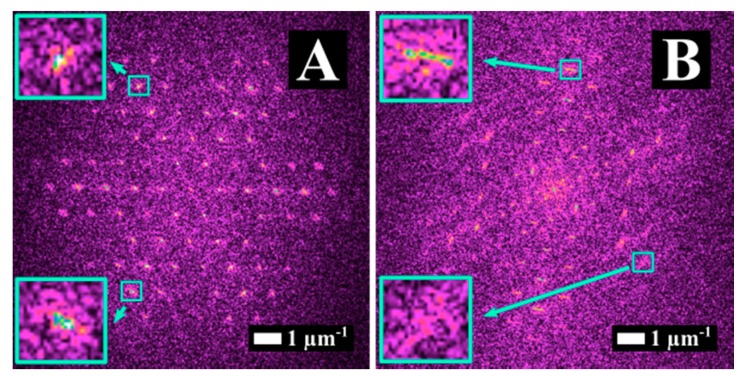
2D-FT graphs of SEM images corresponding to: (**A**) CP pulses, (see Figure 2, Δτ = 3 ps, pps = 20 H = 2 µm); and (**B**) 2D-FT of SEM image relative to XP pulses (see Figure 3, 0.1 J/cm^2^ Δτ = 3 ps, pps = 20 H = 2 µm).

In the specific case of Δτ = 3 ps and Φ_low_, similar structures arose for both CP and XP polarization states. Differently from CP pulses, the results for XP pulses were barely uniform and inhomogeneous. A straightforward confirmation was obtained by comparing the FT analysis of the two cases reported in Figure 4A,B for CP and XP pulses, respectively. In the first case (CP), the peaks were symmetric and sharp, meaning that the structures were homogeneous with a well-defined period along some specific directions. In the second case (XP), the peaks were blurry (see magnification bottom-left) and poorly contrasted with respect to the background, meaning that the period and regularity of the structures were uncertain along all the surface directions.

### 3.2. The Effect of Dose

**Figure 5:**
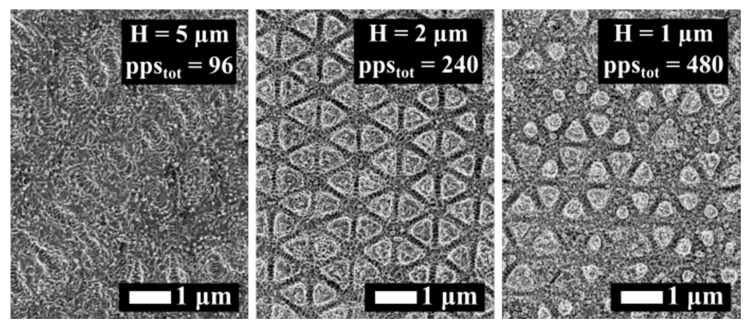
SEM images of stainless steel surface irradiated with CP and different doses, as indicated. Φ = Φ_low_ = 0.1 J/cm^2^, pps = 20 and Δτ = 3 ps.

In Figure 5 we show the surface evolution upon a variation of pps_tot_ for CP when Φ = Φ_low_, pps = 20 and Δτ = 3 ps. For pps_tot_ = 96, the surface morphology consisted of HSFL randomly oriented along the surface and locally organized in a periodic way. For pps_tot_ = 240, prominent well-defined periodic 2D-LIPSS were produced, whereas for pps_tot_ = 480 the surface structures lost their homogeneity. Our results, which are in line with the case of single pulses [13], point out that the triangular structure formation occurs for an optimum pps_tot_ value.

The interplay between Φ and Δτ observed in Figure 2 can be elucidated, considering that an optimum pps_tot_ value leads to a triangular structure formation. Both Δτ [19] and Φ affect the size of the LIPSS textured area. The LIPSS area impacts the effective value of pps_tot_. In that context, the optimum conditions for triangular formation should differ depending on the variation of Φ and Δτ. In detail, a longer Δτ will reduce the effective pps_tot_ value [17,36], whereas a higher Φ value will increase it. This is in accordance with the fact that triangular structures are formed in shorter Δτ for Φ_low_ and in longer Δτ for Φ_med_.

### 3.3. Structure Optimization for Double Pulses

**Figure 6:**
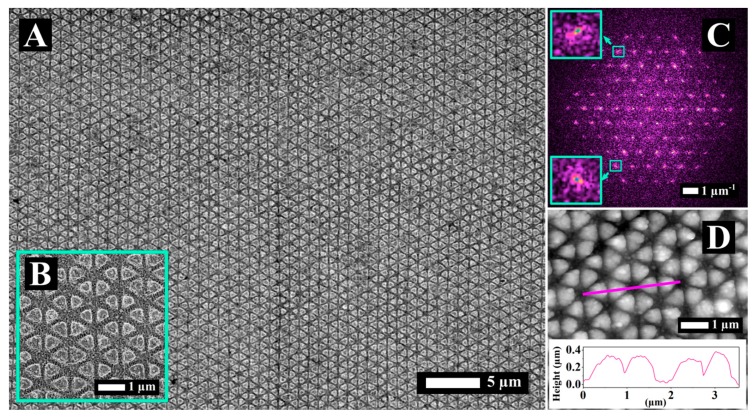
(**A**,**B**) SEM images of 2D structures produced with double XP pulses (Δτ = 3 ps, pps = 10 H = 1 µm and Φ_low_) are shown with two different magnifications. (**C**) 2D-FT of A. (**D**) Profile of the structures shown in A, obtained by AFM analysis carried out along the magenta segment.

In order to optimize the morphology of the triangular textures, we systematically varied pps (pps = 2, 5, 10, 20, 50) and H (H = 1 µm, 2 µm, 5 µm, 10 µm, 20 µm). Figure 6A,B show the SEM images relative to the process parameters of pps = 10 and H = 1 µm processed with XP pulses that enabled an optimum condition. In this case, well defined and homogeneous triangular structures appeared. Furthermore, we extended the process over a surface that was much larger than the spot size. The fact that homogeneous triangular structures can be obtained both with CP (see Table 1) and XP (see Table 2) pulses, for the same delay (Δτ = 3 ps), signifies the predominant role of the delay with respect to the polarization in 2D-LIPSS formation. Moreover, the fact that pulses having different polarization symmetry (XP and CP) lead to the same morphology, within a narrow process window, cannot be interpreted solely with SPP theory [38,39]. In line with works of other groups [30,31,40], we employed the concept of convection flow (CF) to interpret the 2D-LIPSS formation [17]. Considering the occurrence of CF, the formation of the structures was the synergistic result of SPP excitation on the surface during the irradiation and the CF pattern generated by temperature gradients on the surface during the molten phase of the material. In the model of CF [41,42,43], groove pattern formation occurs due to convection roll flow in one (long grooves [16]), two (squares [17]), or three directions (triangular structures [13,17,44]) depending on the excitation conditions. Further experimental validation of the impact of inhomogeneous energy deposition on the microfluidic response of the surface was provided by simultaneous generation of both the direct laser interference pattern and the LIPSS generating 2D microstructures [45]. FT analysis, reported in Figure 6C, reveals a distribution of spatially well-defined peaks with a high contrast with respect to the background. This confirms the regularity of the surface morphology with a high level of order over a large area. From the FT graph we extracted an average structure size of 917 ± 9 nm.

Visualizing the structure profiles was possible via AFM analysis (Figure 6D). A graph of the surface profile is inserted in Figure 6D, corresponding to the magenta line. The structures have an approximately squared profile and the average height (measured for 10 random structures) was found to be 313 ± 46 nm.

### 3.4. Structures Obtained with Bursts of Pulses

Among the advantages of the birefringent crystal setup is that BCs with different lengths can be combined for generating bursts of pulses [32,46]. Such pulse-bursts permit the deposition of a specific amount of energy on the surface, while keeping the single pulse fluence very low. When it comes to LIPSS, the single pulse fluence determines the inhomogeneity of the light absorption [38,47,48], wheras the total fluence determines the melting of the surface and the microfluidic movement [24,31].

Here, we employed this setup to generate bursts of n = 32 sub-pulses and to study the laser-induced formation of structures on the stainless steel surface. Two fluence values were considered, Φ = Φ_low_ and Φ = Φ_high_. Each pulse of the burst had Φ_1/32_ = 0.003 J/cm^2^ in case of Φ_low_ and Φ_1/32_ = 0.013 J/cm^2^ in case of Φ_high_, which is way below the LSFL formation threshold [49]. For single pulse irradiation within the range discussed here (Φ = 0.1–0.4 J/cm^2^), both the amplitude of the inhomogeneous absorption and the dissipated heat were suitable for generating LIPSS [26]. Yet, those conditions were not matched in the case of pulse-burst irradiation. Each pulse fluence was below the damage threshold of the surface and way below the ripple formation threshold [49]. Thus, the inhomogeneous irradiation absorption resulting from a single pulse of the burst cannot enable LIPSS formation. On the contrary, the total fluence of the burst was way above the melting threshold of the material [50]. It has been shown that that for bursts with n = 32 sub-pulses and Δτ = 1.5 ps the melting threshold fluence value is lowered compared to the case of single pulse irradiation [50].

**Figure 7:**
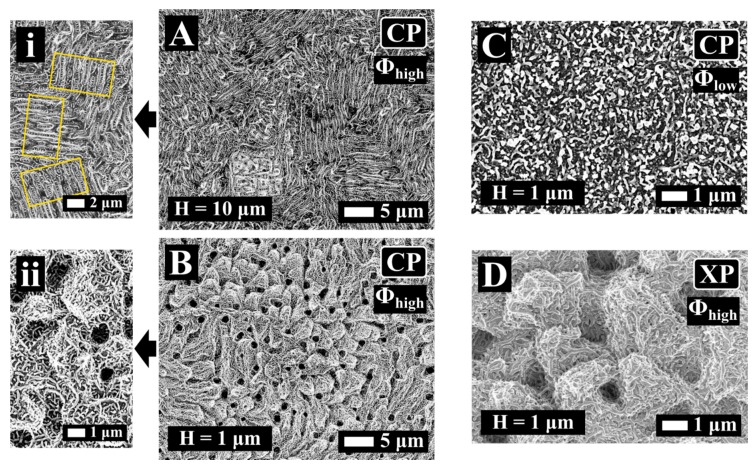
SEM images of stainless steel processed with bursts of n = 32 sub-pulses with Δτ = 1.5 ps. Scanning parameters were: overlap, pps = 20, number of scans N = 5. (**A**) Morphology for H = 10 μm and Φ_burst_ = Φ_high_ in CP, (**B**) morphology for H = 1 µm and Φ_burst_ = Φ_high_ in CP, (**C**) morphology for H = 1 µm and Φ_burst_ = Φ_low_ in CP, and (**D**) morphology for H = 1 µm and Φ_burst_ = Φ_high_ in XP; (**i**) and (**ii**) are zooms of A and B, respectively.

Figure 7 shows SEM images of stainless steel surface after irradiation with bursts of n = 32 sub-pulses with fluence, hatch, and polarization states, as indicated. The surface was scanned five times (N = 5). We varied the hatch to change the pps_tot_ value on the surface. Figure 7A illustrates the morphology for H = 10 μm and Φ_burst_ = Φ_high_. Rippled structures with periods in the range of 1 μm were obtained in this case. Interestingly, the rippled structures were oriented randomly (yellow frames in Figure 7i), in a similar way as HSFL, in Figure 2, for Δτ = 6 ps and Δτ = 12 ps. In the inserted image of higher magnification (Figure 7i), HSFL were visible perpendicular to the ripples with a longer period. 

Upon increasing the dose, when H = 1 μm (Figure 7B), the surface morphology consisted of protrusions of a few micrometers (“micro-hills”) fully covered, with HSFL oriented randomly (Figure 7ii), and together constituting a hierarchical morphology. The polarization did not seem to affect the overall process; the same hierarchical morphology as in Figure 7B was obtained for the same process parameters (H = 1 μm, Φ_high_ and pps = 20) for XP pulse configuration and is shown in Figure 7D. 

For a low fluence value (Φ_burst_ = Φ_low_), employing the same parameters for irradiation (CP, n = 32 and N = 5) the “micro-hills” disappeared and solely nanometric scale features were obtained (Figure 7C). The structures in Figure 7C resemble the HSFL dots obtained for Δτ = 20 ps [18]. The absence of “micro-hills” when Φ_burst_ = Φ_low_ (Figure 7C) indicates that their formation only occured above a certain fluence threshold. Considering that a decrease of the deposited energy results in a thinner melted layer [28], together with the fact that the polarization does not affect their formation mechanism, they should derive solely from the microfluidic motion of the melted surface.

## 4. Conclusions

In this work, we studied the generation of 2D-LIPSS over large areas by irradiating stainless steel surfaces with double-pulses. We employed a robust setup consisting of birefringent crystals for the generation of these double-pulses. Comparing the results obtained in this work with our previous results obtained by a delay-line, we validate the reproducibility of the double-pulse approach, facilitating the 2D-LIPSS generation process. By carrying out a comprehensive study of the main process parameters (inter-pulse delay, pps, fluence, hatch, pps_tot_) we determined an optimum process window that leads to homogeneous 2D-LIPSS over an area much larger than the spot size. In this way, the only limitations come from the used positioning system. Interestingly, triangular 2D-LIPSS formation was optimized independently of the polarization state, CP, or XP, for the same delay (Δτ = 3 ps) and dose (pps_tot_ = 240). In the second step of experiments, a novel supra wavelength 2D-morphology was obtained by pulse-burst irradiation. During this study, we utilized a robust and highly reliable apparatus based on an industrial, high-repetition-rate, high power, femtosecond laser to ensure the scalability of the process. We believe that our results provide a novel insight into the fabrication of 2D-LIPSS and pave the way for this novel surface structuring approach to get a foothold in an industrial environment for high-throughput production.

## Figures and Tables

**Table 1:** Process window for obtaining homogeneous triangular 2D-LIPSS. Comparison between delay line setup and birefringent crystals (BCs) for CP pulses.

Setup	Delay	Overlap (pps)	Hatch (H)	Fluence (Φ)	Dose (pps_tot_)	Source
Delay line	|1 ps|≤ Δτ<|10 ps|	10	1 μm	0.1 J/cm^2^	250	[17]
BCs	1.5 ps, 3 ps	20	2 μm	0.1 J/cm^2^	240	Figure 2

**Table 2:** Process window for obtaining homogeneous triangular 2D-LIPSS. Comparison between delay-line setup and birefringent crystals (BCs) for XP pulses.

Setup	Delay	Overlap (pps)	Hatch (H)	Fluence (Φ)	Dose (pps_tot_)	Source
Delay line	|1 ps|≤ Δτ<|5 ps|	10	1 μm	0.1 J/cm^2^	250	[17]
BCs	3 ps	10	1 μm	0.1 J/cm^2^	240	Figure 6

## References

[1] Zorba V., Stratakis E., Barberoglou M., Spanakis E., Tzanetakis P., Anastasiadis S.H., Fotakis C. (2008). Biomimetic artificial surfaces quantitatively reproduce the water repellency of a lotus leaf. Adv. Mater..

[2] Vorobyev A.Y., Guo C. (2008). Colorizing metals with femtosecond laser pulses. Appl. Phys. Lett..

[3] Guay J.-M., Calà Lesina A., Côté G., Charron M., Poitras D., Ramunno L., Berini P., Weck A. (2017). Laser-induced plasmonic colours on metals. Nat. Commun..

[4] Bonse J., Koter R., Hartelt M., Spaltmann D., Pentzien S., Höhm S., Rosenfeld A., Krüger J. (2015). Tribological performance of femtosecond laser-induced periodic surface structures on titanium and a high toughness bearing steel. Appl. Surf. Sci..

[5] Epperlein N., Menzel F., Schwibbert K., Koter R., Bonse J., Sameith J., Krüger J., Toepel J. (2017). Influence of femtosecond laser produced nanostructures on biofilm growth on steel. Appl. Surf. Sci..

[6] Simitzi C., Efstathopoulos P., Kourgiantaki A., Ranella A., Charalampopoulos I., Fotakis C., Athanassakis I., Stratakis E., Gravanis A. (2015). Laser fabricated discontinuous anisotropic microconical substrates as a new model scaffold to control the directionality of neuronal network outgrowth. Biomaterials.

[7] Xia F., Jiang L. (2008). Bio-Inspired, Smart, Multiscale Interfacial Materials. Adv. Mater..

[8] Elbourne A., Crawford R.J., Ivanova E.P. (2017). Nano-structured antimicrobial surfaces: From nature to synthetic analogues. J. Colloid Interface Sci..

[9] Siddique R.H., Gomard G., Hölscher H. (2015). The role of random nanostructures for the omnidirectional anti-reflection properties of the glasswing butterfly. Nat. Commun..

[10] Bonse J., Hohm S., Kirner S.V., Rosenfeld A., Kruger J. (2017). Laser-Induced Periodic Surface Structures—A Scientific Evergreen. IEEE J. Sel. Top. Quantum Electron..

[11] Romano J., Helbig R., Fraggelakis F., Garcia-Giron A., Werner C., Kling R., Dimov S. (2019). Springtail-inspired triangular laser-induced surface textures on metals using MHz ultrashort pulses. Accept. ASME J. Micro Nano Manuf..

[12] Skoulas E., Manousaki A., Fotakis C., Stratakis E. (2017). Biomimetic surface structuring using cylindrical vector femtosecond laser beams. Sci. Rep..

[13] Romano J.-M., Garcia-Giron A., Penchev P., Dimov S. (2018). Triangular laser-induced submicron textures for functionalising stainless steel surfaces. Appl. Surf. Sci..

[14] Gregorčič P., Sedlaček M., Podgornik B., Reif J. (2016). Formation of laser-induced periodic surface structures (LIPSS) on tool steel by multiple picosecond laser pulses of different polarizations. Appl. Surf. Sci..

[15] Cong J., Yang J., Zhao B., Xu X. (2015). Fabricating subwavelength dot-matrix surface structures of Molybdenum by transient correlated actions of two-color femtosecond laser beams. Opt. Express.

[16] Fraggelakis F., Mincuzzi G., Lopez J., Manek-Hönninger I., Kling R. (2018). 2D laser induced periodic surface structures with double cross-polarized pulses. Laser-Based Micro-Nanoprocessing XII.

[17] Fraggelakis F., Mincuzzi G., Lopez J., Manek-Hönninger I., Kling R. (2019). Controlling 2D laser nano structuring over large area with double femtosecond pulses. Appl. Surf. Sci..

[18] Fraggelakis F., Mincuzzi G., Lopez J., Kling R., Manek-Hönninger I. (2018). Controlling Micron and Submicron Scale Laser Induced Surface Structures on Stainless Steel with Industrial Femtosecond Lasers. J. Laser Micro/Nanoeng..

[19] Derrien T.J.-Y.J.Y., Krüger J., Itina T.E., Höhm S., Rosenfeld A., Bonse J. (2013). Rippled area formed by surface plasmon polaritons upon femtosecond laser double-pulse irradiation of silicon: the role of carrier generation and relaxation processes. Appl. Phys. A Mater. Sci. Process..

[20] Giannuzzi G., Gaudiuso C., Di Franco C., Scamarcio G., Lugarà P.M., Ancona A. (2019). Large area laser-induced periodic surface structures on steel by bursts of femtosecond pulses with picosecond delays. Opt. Lasers Eng..

[21] Höhm S., Herzlieb M., Rosenfeld A., Krüger J., Bonse J. (2015). Dynamics of the formation of laser-induced periodic surface structures (LIPSS) upon femtosecond two-color double-pulse irradiation of metals, semiconductors, and dielectrics. Appl. Surf. Sci..

[22] Fraggelakis F., Stratakis E., Loukakos P.A. (2018). Control of periodic surface structures on silicon by combined temporal and polarization shaping of femtosecond laser pulses. Appl. Surf. Sci..

[23] Sundaram S.K., Mazur E. (2002). Inducing and probing non-thermal transitions in semiconductors using femtosecond laser pulses. Nat. Mater..

[24] Tsibidis G.D., Barberoglou M., Loukakos P.A., Stratakis E., Fotakis C. (2012). Dynamics of ripple formation on silicon surfaces by ultrashort laser pulses in subablation conditions. Phys. Rev. B.

[25] Déziel J.-L., Dumont J., Gagnon D., Dubé L.J., Messaddeq S.H., Messaddeq Y. (2015). Toward the formation of crossed laser-induced periodic surface structures. J. Opt..

[26] Tsibidis G.D., Mimidis A., Skoulas E., Kirner S.V., Krüger J., Bonse J., Stratakis E. (2018). Modelling periodic structure formation on 100Cr6 steel after irradiation with femtosecond-pulsed laser beams. Appl. Phys. A.

[27] Garcia-Lechuga M., Puerto D., Fuentes-Edfuf Y., Solis J., Siegel J. (2016). Ultrafast Moving-Spot Microscopy: Birth and Growth of Laser-Induced Periodic Surface Structures. ACS Photonics.

[28] Zhigilei L.V., Lin Z., Ivanov D.S. (2009). Atomistic modeling of short pulse laser ablation of metals: Connections between melting, spallation, and phase explosion. J. Phys. Chem. C.

[29] Fang R., Vorobyev A., Guo C. (2017). Direct visualization of the complete evolution of femtosecond laser-induced surface structural dynamics of metals. Light Sci. Appl..

[30] Tsibidis G.D., Skoulas E., Papadopoulos A., Stratakis E. (2016). Convection roll-driven generation of supra-wavelength periodic surface structures on dielectrics upon irradiation with femtosecond pulsed lasers. Phys. Rev. B.

[31] Kirichenko N.A., Barmina E.V., Shafeev G.A. (2018). Theoretical and Experimental Investigation of the Formation of High Spatial Frequency Periodic Structures on Metal Surfaces Irradiated by Ultrashort Laser Pulses. Phys. Wave Phenom..

[32] Zhou S., Ouzounov D., Li H., Bazarov I., Dunham B., Sinclair C., Wise F. (2007). Efficient temporal shaping of ultrashort pulses with birefringent crystals. Appl. Opt..

[33] Rapp S., Kaiser M., Schmidt M., Huber H.P. (2016). Ultrafast pump-probe ellipsometry setup for the measurement of transient optical properties during laser ablation. Opt. Express.

[34] Barberoglou M., Tsibidis G.D., Gray D., Magoulakis E., Fotakis C., Stratakis E., Loukakos P.A. (2013). The influence of ultra-fast temporal energy regulation on the morphology of Si surfaces through femtosecond double pulse laser irradiation. Appl. Phys. A Mater. Sci. Process..

[35] Hoöhm S., Rohloff M., Rosenfeld A., Kruger J., Bonse J. (2013). Dynamics of the formation of laser-induced periodic surface structures on dielectrics and semiconductors upon femtosecond laser pulse irradiation sequences. Appl. Phys. A Mater. Sci. Process..

[36] Höhm S., Rosenfeld A., Krüger J., Bonse J. (2013). Area dependence of femtosecond laser-induced periodic surface structures for varying band gap materials after double pulse excitation. Appl. Surf. Sci..

[37] Sedao X., Maurice C., Garrelie F., Colombier J.P., Reynaud S., Quey R., Pigeon F. (2014). Influence of crystal orientation on the formation of femtosecond laser-induced periodic surface structures and lattice defects accumulation. Appl. Phys. Lett..

[38] Bonse J., Rosenfeld A., Krüger J. (2009). On the role of surface plasmon polaritons in the formation of laser-induced periodic surface structures upon irradiation of silicon by femtosecond-laser pulses. J. Appl. Phys..

[39] Sipe J.E., Young J.F., Preston J.S., Van Driel H.M. (1983). Laser-induced periodic surface structure. I. Theory. Phys. Rev. B.

[40] Tsibidis G.D., Fotakis C., Stratakis E. (2015). From ripples to spikes: A hydrodynamical mechanism to interpret femtosecond laser-induced self-assembled structures. Phys. Rev. B.

[41] Koschmieder E.L., Pallas S.G. (1974). Heat transfer through a shallow, horizontal convecting fluid layer. Int. J. Heat Mass Transf..

[42] Busse F.H. (1978). Non-linear properties of thermal convection. Reports Prog. Phys..

[43] Cross M.C., Hohenberg P.C. (1993). Pattern formation outside of equilibrium. Rev. Mod. Phys..

[44] Liu Q., Zhang N., Yang J., Qiao H., Guo C. (2018). Direct fabricating large-area nanotriangle structure arrays on tungsten surface by nonlinear lithography of two femtosecond laser beams. Opt. Express.

[45] Alamri S., Fraggelakis F., Kunze T., Krupop B., Mincuzzi G., Kling R., Lasagni A.F. (2019). On the Interplay of DLIP and LIPSS Upon Ultra-Short Laser Pulse Irradiation. Materials.

[46] Dromey B., Zepf M., Landreman M., O’Keeffe K., Robinson T., Hooker S.M. (2007). Generation of a train of ultrashort pulses from a compact birefringent crystal array. Appl. Opt..

[47] Skolski J.Z.P., Römer G.R.B.E., Obona J.V., Ocelik V., Huis In ’t Veld A.J., De Hosson J.T.M. (2012). Laser-induced periodic surface structures: Fingerprints of light localization. Phys. Rev. B.

[48] Skolski J.Z.P., Römer G.R.B.E., Vincenc Obona J., Huis in ’T Veld A.J. (2014). Modeling laser-induced periodic surface structures: Finite-difference time-domain feedback simulations. J. Appl. Phys..

[49] Bonse J., Hoöhm S., Rosenfeld A., Kruger J. (2013). Sub-100-nm laser-induced periodic surface structures upon irradiation of titanium by Ti:sapphire femtosecond laser pulses in air. Appl. Phys. A Mater. Sci. Process..

[50] Gaudiuso C., Giannuzzi G., Volpe A., Lugarà P.M., Choquet I., Ancona A. (2018). Incubation during laser ablation with bursts of femtosecond pulses with picosecond delays. Opt. Express.

